# Firearm injury hospitalizations and handgun purchaser licensing laws: longitudinal evaluation of state-level purchaser licensure requirements on firearm violence, 2000–2016

**DOI:** 10.1186/s40621-024-00522-4

**Published:** 2024-08-23

**Authors:** Mitchell L. Doucette, Nicholas S. Meyerson, Cassandra K. Crifasi, Elizabeth Wagner, Daniel W. Webster

**Affiliations:** Center for Gun Violence Solutions, Johns Hopkins Bloomberg School of Public Health, 624 North Broadway, Baltimore, MD 21205 USA

## Abstract

**Background:**

Handgun purchaser licensing (HPL) laws mandate individuals to obtain a license from law enforcement before buying a firearm. Research indicates these laws effectively reduce various forms of fatal firearm violence, including homicides, suicides, and mass shootings. Our study sought to assess the impact of HPL laws on non-fatal firearm violence.

**Methods:**

Utilizing the augmented synthetic control method (ASCM), we estimated the average treatment effect on the treated (ATT) resulting from a full repeal of an HPL law in Missouri (2007), a partial repeal in Michigan (2012), and an adoption on HPL law in Maryland (2013) on firearm injury hospitalizations. We utilized RAND's healthcare cost and utilization project-based dataset from 2000 to 2016 for our outcome variable. We conducted in-time placebo testing and leave-one-out donor pool testing as sensitivity analyses.

**Results:**

Maryland’s adoption was associated with a statistically significant 32.3% reduction in firearm-related injury hospitalization (FIH) rates (ATT = − 0.497, standard error (SE) = 0.123); Missouri’s repeal was associated with a statistically significant 35.7% increase in FIH rates (ASCM = 0.456, SE = 0.155); and Michigan’s partial repeal showed no statistically significant associations with FIH rates (ATT = − 0.074, SE = 0.129). Sensitivity analyses confirm the robustness of the estimated HPL effects.

**Discussion:**

HPL laws appear to be protective against hospitalizations for nonfatal firearm injuries. These findings align with prior research indicating that HPL laws are effective in reducing fatal firearm violence. States without such licensing systems ought to consider these robust policies as a means to address firearm violence.

**Supplementary Information:**

The online version contains supplementary material available at 10.1186/s40621-024-00522-4.

## Introduction

Gun violence, both homicide and suicide, is one of the prevailing public health concerns in the United States. In total, 48,830 Americans died from firearm injuries in 2021, with 53.9% from suicide and 42.9% from homicide (CDC 2021). In a given year, more than twice the number of people who are killed with a firearm are injured; most of these injuries are assaults followed by unintentional injuries. Research suggests the total cost of firearm injuries was $493.2 billion dollars in 2020 in the United States (Miller et al. 2019).

Federal laws require prospective firearm purchasers to complete an application at the point of purchase when buying firearms from Federal Firearm Licensees (FFLs) (i.e., licensed dealers) (Brady 2023). FFLs submit purchase applications either electronically or by phone to the Federal Bureau of Investigation's (FBI) National Instant Criminal Background Check System to complete a background check. This process normally takes minutes; however, in some cases, it can take a maximum of three business days. After three days, if the background check has not been completed, the default is to proceed with the sale. Under federal law, there are no regulations for firearm transfers by private individuals. Twenty-one states and the District of Columbia have laws that extend background check requirements for handgun purchasers to most private transactions (Giffords Law Center 2023).

The Brady Handgun Violence Prevention Act, enacted in 1993 and effective in 1994, required Federally-licensed firearms dealers to make firearms transfers contingent upon prospective transferees passing a background check (Bureau of Alcohol, Tobacco, Firearms, and Explosives 2021). Permanent implementation of the Brady Act established the National Instant Criminal Background Check System. This system facilitates efficient submission of applications to acquire firearms and determinations by the FBI. Importantly, the Brady Act background check requirements did not apply to firearm transfers by someone who was not a licensed firearms dealer.

Handgun purchaser licensing (HPL) laws extend federal regulations further mandating individuals seeking to buy a handgun to apply to state or local police for a permit or license before purchase, regardless of seller type (Crifasi et al. 2019; Johns Hopkins Center for Gun Violence Solutions 2024). The process includes a thorough background check accessing local, state, and federal records, often with mandated fingerprinting. HPLs include a waiting period for new purchasers, allowing time for background checks. These laws aim to improve background check accuracy, facilitate vetting by private sellers, and deter illegal straw purchases.

Eight states, Connecticut, Hawaii, Illinois, Maryland, Massachusetts, Nebraska, New Jersey, and New York, and the District of Columbia currently have a HPL law. Oregon passed an HPL law via ballot initiative in 2022 but that law has not yet been implemented due to legal challenges. North Carolina and Iowa repealed their HPL laws in 2023 and 2021 respectively (Schoenbaum 2023; Iowa Department of Public Safety 2024). Only Connecticut (1995) and Maryland (2013) have adopted HPL laws within the past 30 years that are currently active. Missouri repealed its long standing HPL law in 2007. Michigan partially repealed its HPL law in 2013, removing the requirement for an HPL for FFL transactions. In 2024, Michigan subsequently adopted legislation restoring their HPL law to apply to all firearm transactions (Giffords Law Center 2024).

Research on the impact of HPL laws on firearm violence has investigated the relationship between law changes and intermediate outcomes such as diversion of firearms for criminal use (Crifasi et al. 2013; Webster and Vernick 2013) and long-term outcomes such as firearm homicides (Rudolph et al. 2015; Hasegawa et al. 2019; McCourt et al. 2020; Webster et al. 2014), suicides (McCourt et al. 2020; Crifasi et al. 2015), and mass shootings (Siegel et al. 2020; Webster et al. 2020). Prohibited individuals most commonly acquire firearms through straw purchases or other sales with private, unlicensed sellers (Braga et al. 2012). From a causal perspective, the robust application and screening process of a HPL law makes illicit firearm acquisition more difficult (Webster and Vernick 2013). A survey of individuals with experience in Baltimore’s underground firearm market found 40% of anonymous respondents reported having a more difficult time finding a firearm after the 2013 HPL law adoption (Crifasi et al. 2013). The more in-depth licensing process also likely reduces access to a highly lethal means of suicide.

Downstream effects support these hypotheses, as the implementation of a HPL in Connecticut in 1995 saw lower rates of homicides and suicides (Rudolph et al. 2015; McCourt et al. 2020; Crifasi et al. 2015). Other results suggest the inverse is true, with increased rates of homicides and suicides in Missouri after the state repealed their HPL in 2007 (Hasegawa et al. 2019; McCourt et al. 2020; Crifasi et al. 2015; Li et al. 2023; Bhatt et al. 2020). Another state-level analysis found states with HPL laws had lower rates of mass-shootings from 1976 to 2018 (Siegel et al. 2020). County-level studies of large metropolitan areas found similar associations, with the presence of a HPL law associated with lower rates of firearm homicides (Doucette et al. 2021; Crifasi et al. 2018).

### Current contribution

To date, nearly all of the research examining the effect of HPL laws has focused on fatal firearm violence. Therefore, we sought to examine the impact of HPL laws on non-fatal firearm injuries using inpatient hospitalization data. We hypothesized that states that adopted HPL laws saw lower rates of non-fatal firearm violence while states that repealed their HPL laws saw higher rates.

## Methods

We used a quasi-experimental design to estimate the association between the adoption and repeal of HPL laws on firearm inpatient hospitalization (FIH) rates per 100,000 population. We estimated the average treatment effect on the treated resulting from the law changes using a comparative time-series analysis, the augmented synthetic control method (ASCM).

### Data and variables

For our outcome, we used a publicly available dataset of FIH rates from 2000 to 2016 published by the RAND Corporation (Smart et al. 2000). Comprehensive, national-level data related to non-fatal firearm injuries that result in a hospitalization within the United States were not collected. Some states collect this information, referred to as State Inpatient Databases (SID), and shared it with the Agency for Healthcare Research and Quality’s (AHRQ) Healthcare Cost and Utilization Project (H-CUP). However, this data, as a resource for national-level analyses, suffers from several limitations (Smart et al. 2000). Several states do not report SIDs to H-CUP or only recently begun indexing relevant hospitalization records into a SID. There is potential missingness also associated with SIDs particularly for aspects of medical records needed for identifying injuries resulting from a firearm discharge (Smart et al. 2000).

In an attempt to compile a useful national-level dataset of firearm injuries resulting in a hospitalization, the RAND corporation aggregated multiple SID data sources from 2000 to 2016 (Smart et al. 2000). RAND used the SID available through H-CUP and further supplemented this information from state-specific SID data as well as state health department data. The data was cleaned and imputed using a variety of covariates hypothesized as associated with firearm hospitalizations rates. The researchers used a single Bayesian regression model with a complex error structure. The result is a publicly available dataset that provides a balanced panel of inpatient FIH rates for each state over the study period. Rates were not stratified by victim demographic or intent (i.e., homicides or suicides). Please see the published report by Smart, Peterson, Schell, and colleagues (2021) for further information (Smart et al. 2000).

In our analysis, we included the following time-varying covariates, each measured in terms of the percentage of the state population: male, veteran, married, living in poverty, and living in a metropolitan statistical area. Additionally, we incorporated per capita personal income, ethanol consumption rate, and number of law enforcement officers employed. Covariates were sourced from the U.S. Census Bureau, the Bureau of Labor Statistics, the National Institute on Alcohol Abuse and Alcoholism, the Bureau of Justice Statistics, and the Uniform Crime Reports, respectively. We selected these covariates based on their known associations with firearm violence (Doucette et al. 2021; Crifasi et al. 2018; Wintemute 2015a, b; Krivo et al. 2009; Kalesan et al. 2014; Jacoby et al. 2018).

### Treatment and Control Units

The study spanned from 2000 to 2016, a period during which Missouri completely repealed its Handgun Purchaser Licensing (HPL) law in 2007 and Michigan modified its HPL law in 2012 by removing the HPL requirement for purchases from FFLs. To construct the counterfactual for the impact of these legal changes, we compared states that had repealed their HPL laws with those that had retained them throughout the duration of the study. Nine control units qualified for inclusion as a basis for comparison: Connecticut, Hawaii, Illinois, Iowa, Massachusetts, Nebraska, New Jersey, New York, and North Carolina. Despite Connecticut adopting an HPL in 1995, we included it as a control state as the law had been in effect for over 10 years before Missouri's repeal. In the analyses of repeals, Missouri had 9 years of post-repeal data (2008–2016), and Michigan had 4 years (2013–2016).^1^

Maryland adopted an HPL law in 2013. To construct the counter-factual for HPL law adoption, a total of 38 states that had never adopted HPL laws were used as a comparison.^2^ In these analyses, Maryland had 3 years of post-implementation data (2014–2016). We obtained state HPL law effective dates via our own legal research and verified them against public databases of state firearm laws and prior research on concealed carry laws (Giffords Law Center 2022; Everytown for Gun Safety 2022). We provided the average FIH rates per 100,000 pre-and-post law changes comparing treatment states to their controls. All law change years were lagged one year to the first year of implementation.

### Data analysis

The augmented synthetic control method (ASCM), an extension of the synthetic control method (SCM) by Ben-Michael et al. (2021), builds on the SCM introduced by Abadie and Gardeazbal (2003), widely recognized for its robustness in measuring policy impacts. SCM creates a 'synthetic state' representing the pre-intervention trend of the treated state before a policy change. This synthetic state is constructed using non-negative weights from a donor pool of comparison states potentially affected by the policy change. Donor state weights are determined based on lagged outcome values and covariates, minimizing the discrepancy between the treated state and the synthetic state before the policy change (Abadie 2021, 2003; Abadie et al. 2014). The root mean square error (RMSE) assesses the assumption of equivalence between the counterfactual and observed outcomes in the pre-trend period. Therefore, a model's performance in accurately representing the treatment's counterfactual depends on its RMSE during this period.

The ASCM method builds upon this method by introducing a linear regression model, regressing the weighted synthetic state on the treatment (Ben-Michael et al. 2021). The linear regression utilizes a ridge estimate, rather than a least square estimator, and introduces a L2-norm penalty term. The augmented version of the Synthetic Control Method offers more precise estimation of standard errors, particularly useful in addressing multicollinearity concerns. Unlike SCM, ASCM’s flexible weighting allows for negative weights. ASCM has recently been used to examine opioids use (McGinty et al. 2022) firearm violence (Doucette et al. 2022a, b, 2023), and pollution, among other public health concerns (Cole et al. 2020). For this analysis, to ensure the best counterfactuals were used for estimating the average treatment effect on the treated related to changes to HPL laws, we compared model performance of the SCM and the ASCM via the RMSE. It should be noted that dichotomous policy variables were excluded from this analysis, as SCM and ASCM methodologies cannot derive weights from time-invariant factors. We calculated the percent increase or decrease related to HPL law change by dividing the average treatment effect on the treated by the counterfactual post-treatment average FIH rate (i.e., the actual post-treatment trend minus the average treatment effect on the treated) and multiplying by 100.

#### Sensitivity analyses

We performed two sensitivity analyses to assess the robustness of the findings. First, we conducted basic in-place placebo testing. This method estimates the average treatment effect on the treated for each of the donor states as if they were the treatment state. A comparison of all the average treatment effect on the treated estimates then provides context for whether the true treatment state’s average treatment effect on the treated is part of a larger trend or aberrant, indicating the likelihood that the original finding is directly related to the policy change. However, this method can be prone to error due to the ASCMs inability to control for policy variables. As a complement, we also conducted leave-one-out sensitivity analyses. For each treatment state, we estimated the average treatment effect on the treated and standard error of the HPL law change *n* − 1 times, where *n* = the number of controls within the treatment state’s donor pool. For each model, we left out 1 donor state to test how stable the average treatment effect on the treated and standard error was across varying donor pools and compared it to the full donor pool model.

## Results

We examined the mean changes in FIH rates per 100,000 before and after HPL changes, as presented in Table 1. Missouri's FIH rate increased by 0.195 (11.9%), whereas the average of its control states decreased by 0.41 (5.5%). Michigan’s FIH rate decreased by 0.289 (− 22.3%), and its control states also experienced a decrease of 0.091 (− 12.7%). Maryland's FIH rate showed a notable decrease of 0.675 (− 48.9%), while its control states experienced a more modest decrease of 0.015 (− 1.6%). All treatment states exhibited significantly higher FIH rates in the pre-treatment period compared to the average of their control states.

**Table 1:** Average firearm inpatient hospitalization rate per 100,000, prior to and after law changes compared to their respective control groups

	Prior to law change	After law change	Difference
*Missouri*	1.537	1.732	0.195
Controls	0.765	0.724	− 0.041
*Michigan*	1.439	1.15	− 0.289
Controls	0.765	0.674	− 0.091
*Maryland*	1.719	1.044	− 0.675
Controls	0.938	0.923	− 0.015

Missouri repealed its HPL law in 2007, Michigan partially repealed its HPL law in 2012, and Maryland adopted its HPL law in 2013. Missouri and Michigan control states consisted of Connecticut, Hawaii, Illinois, Iowa, Massachusetts, Nebraska, New Jersey, New York, and North Carolina; Maryland control states consisted of all other states excluding the control states for Missouri and Michigan

**Fig. 1:**
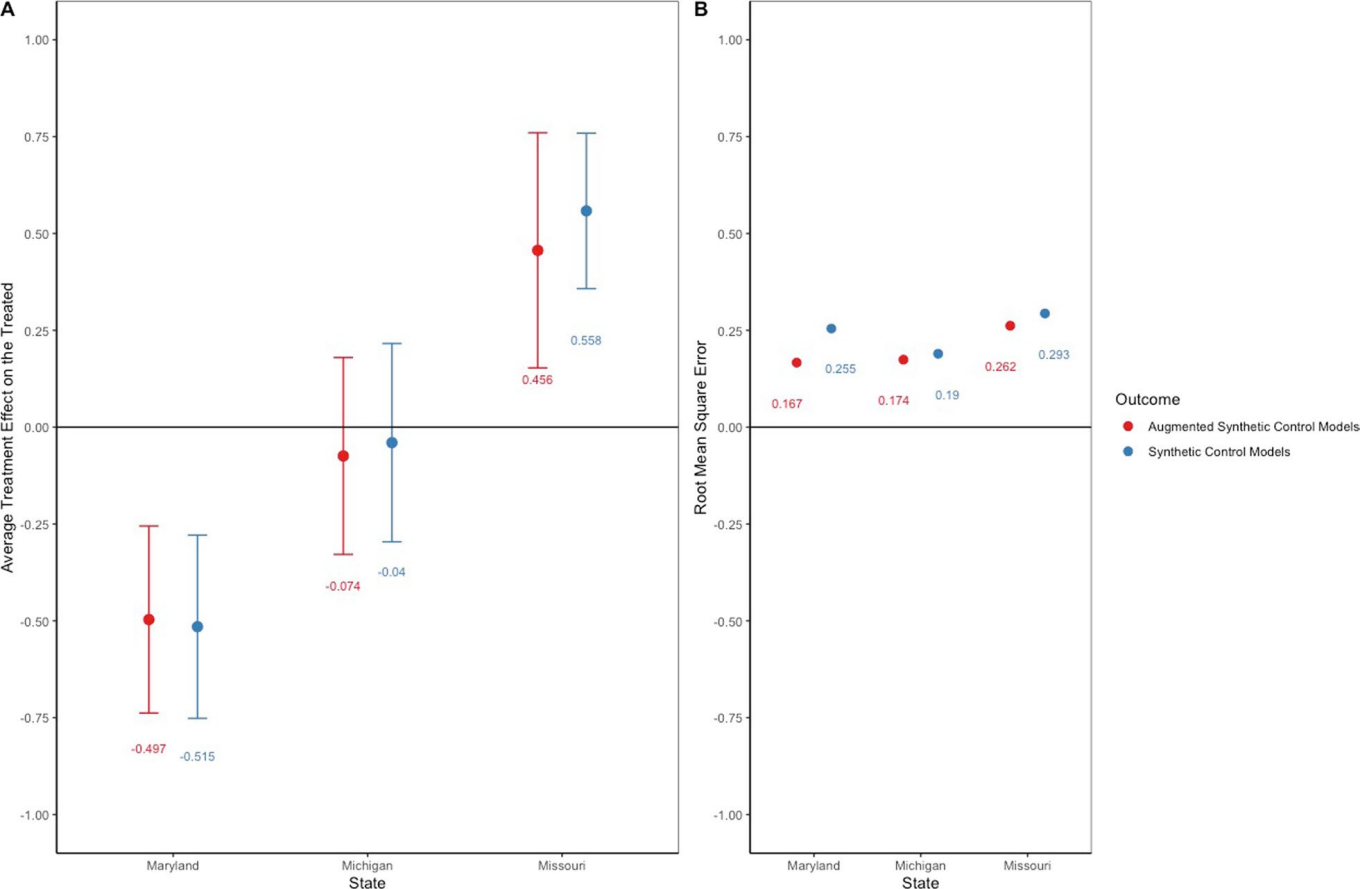
Average treatment effect on the treated and standard error (Plot A) as well as root mean square prediction error (Plot B) associated with changes to handgun purchaser license laws for Maryland, Michigan, and Missouri. Values presented for synthetic control models and augmented synthetic control models. Error bars are jackknife standard errors

We utilized ASCM and SCM to estimate the average treatment effect on the treated from changes in HPL laws on FIH rates. Figure 1 illustrates the average treatment effect on the treated and its standard error, along with the RMSE for both models—SCM in blue and ASCM in red. The ASCM and SCM analyses yielded similar average treatment effect on the treated estimates and RMSEs within states. HPL law adoption was associated with lower FIH rates in Maryland (ASCM average treatment effect on the treated = − 0.497, standard error = 0.123; SCM average treatment effect on the treated = − 0.515, standard error = 0.121). The repeal of an HPL was associated with increased FIH rates in Missouri (ASCM average treatment effect on the treated = 0.456, standard error = 0.155; SCM average treatment effect on the treated = 0.558, standard error = 0.102). Michigan's partial repeal did not exhibit significant associations with changes in FIH rates (ASCM average treatment effect on the treated = − 0.074, standard error = 0.129; SCM average treatment effect on the treated = − 0.039, standard error = 0.131). The average treatment effect on the treated from the ASCM for HPL adoption in Maryland was estimated to have a 32.3% reduction in FIH rates. HPL repeal in Missouri was estimated to have a 35.7% increase, and partial repeal in Michigan was estimated to have a 6.1% decrease in FIH rates.

The pre-treatment values for each treatment state and their augmented synthetic controls seem to be well balanced (refer to Supplemental Tables 1–3). As seen in Table 1, our treatment states all had comparatively higher rates of FIH than their pool of controls and suggests the pre-treatment fit of the ASCM models have balanced well on the outcome and covariates. Also found in Supplemental Tables 1–3, we presented the mean pre-treatment covariate values for each donor state, donor weights, and the mean ASCM covariate values for each state. North Carolina had the largest donor weight for Missouri (0.74), Illinois for Michigan (0.58), and Virginia for Maryland (0.51).

**Fig. 2:**
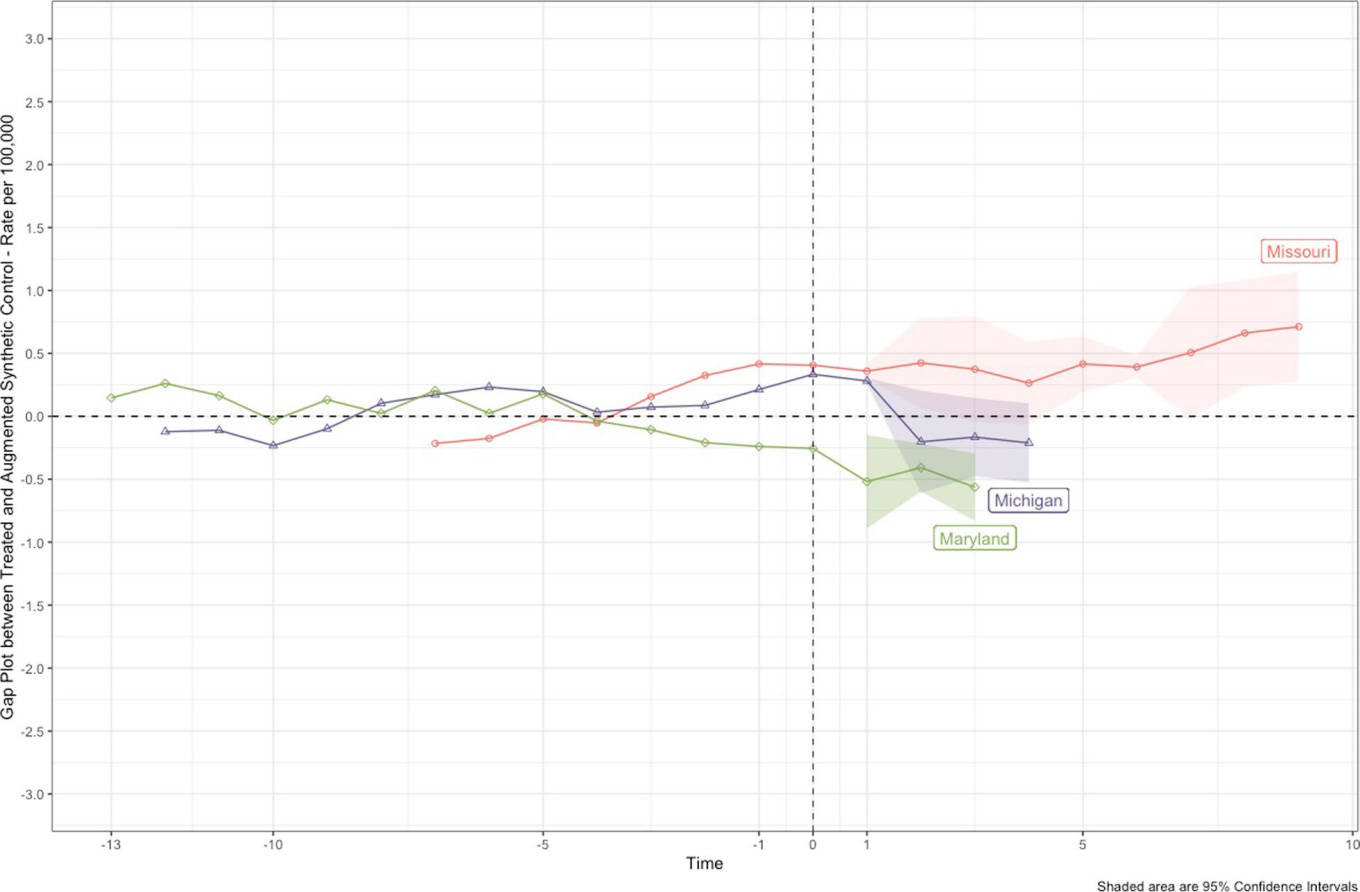
Combined Gap plots for augmented synthetic control models estimating effect of handgun purchaser license law changes on firearm inpatient hospitalization rates, 2000–2016. Error bars are jackknife standard errors

We provided gap plots, displaying the differences between the treated state and their synthetic control state, for each state in Fig. 2. For Missouri, the difference in the pre-treatment trajectory showed a slight upward trend in the years leading up to treatment; however, there are no significant spikes, and the RMSE value is small (RMSE = 0.262) (RMSE values are provided in Fig. 1). The same pattern held for Maryland, albeit in the opposite direction. There was a slight downward trend in the pre-treatment period for Maryland, but without any significant year-over-year spikes. It exhibited a small RMSE value (RMSE = 0.167), indicating an adequate fit for inference. Michigan appeared to be the most stable among the three as it displayed some slight fluctuations over time and a small RMSE value of 0.174.

There are potential issues with extrapolation that may arise when ASCMs contain vary large weights and/or a large proportion of donors with negative weights (See Supplemental Tables 1–3) (Abadie and Vives-i-Bastida 2022). As such, we conducted an ad hoc sensitivity test wherein we demeaned our outcome by panel and re-ran our models. We provided the results of this ad hoc test of internal validity in Supplemental Table 4. We saw that our primary models and the demeaned models produce nearly the same average treatment effects on the treated and standard errors.

**Fig. 3:**
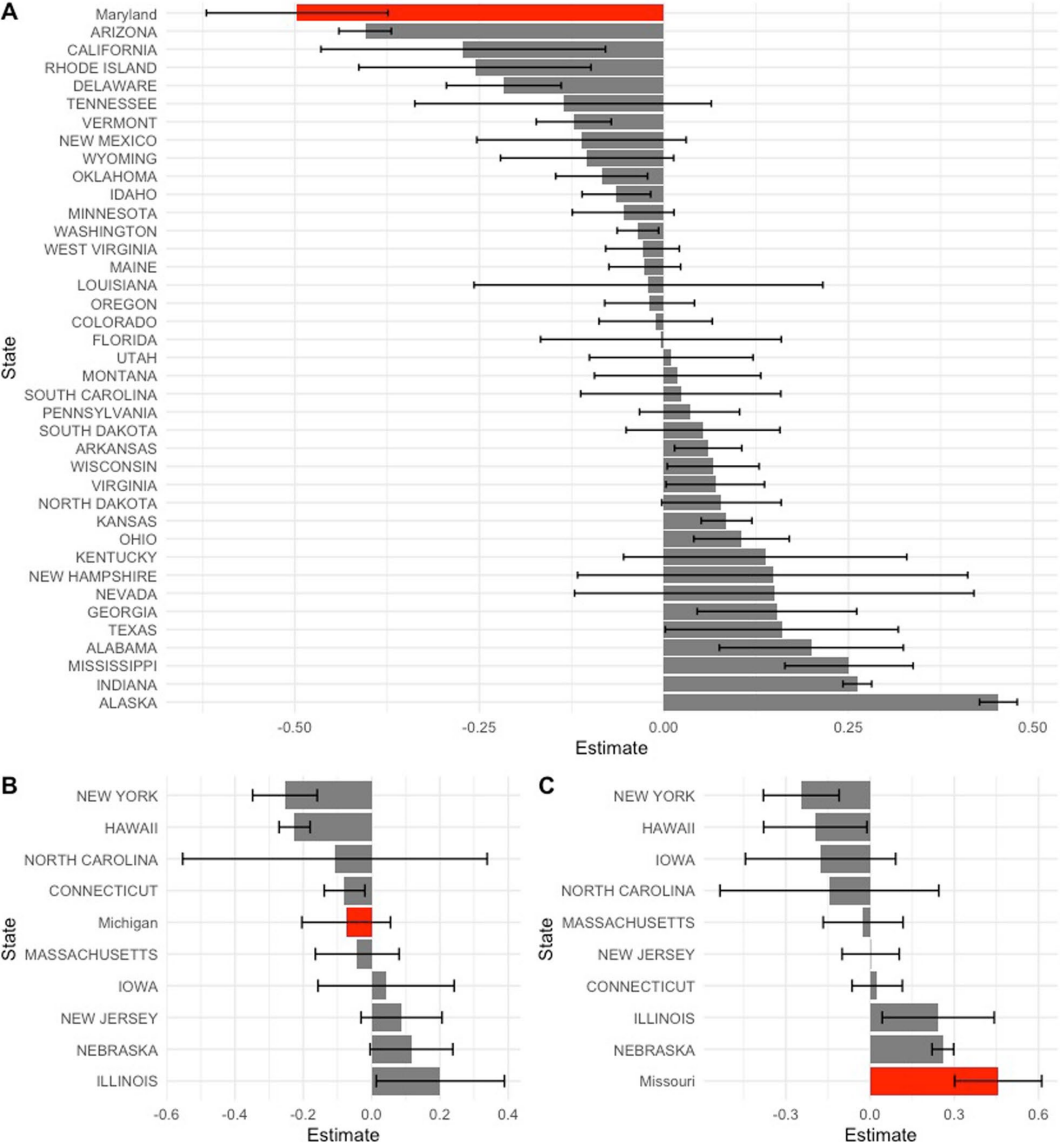
In-place placebo testing for impact of change to handgun purchaser licensing laws on firearm inpatient hospitalization rates per 100,000. Model is augmented synthetic control models. Estimate is average treatment effect on the treated and error bars are jackknife standard errors. A = Maryland, B = Michigan, and C = Missouri. Treatment states highlighted in red

We presented the first of two sensitivity analyses examining the robustness of our findings in Fig. 3. First, we conducted an in-place placebo testing related to the impact of HPL law changes on FIH rates per 100,000. In these analyses, each control state was treated as if it were the true adopting state, relegating the true treatment state to a control. It is noteworthy that the estimated average treatment effect on the treated and standard error for the Maryland model represents the largest reduction compared to each of the placebo controls. We also observed that Michigan’s estimated average treatment effect on the treated and standard error were positioned near the middle of all placebo controls, displaying non-significance. Missouri exhibited the largest increase compared to the other placebo controls.

**Fig. 4:**
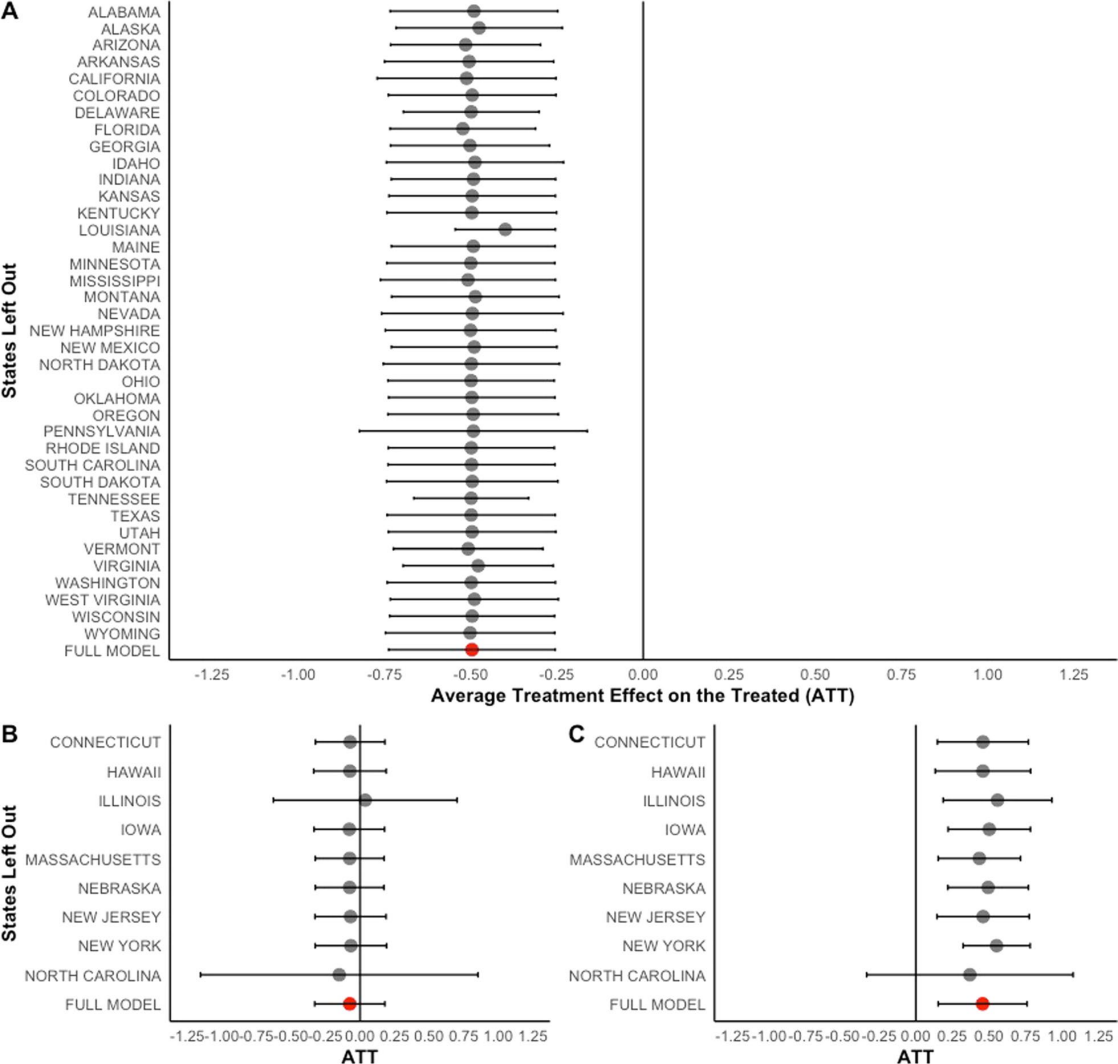
Leave-one-out sensitivity analysis for impact of change to handgun purchaser licensing laws on firearm inpatient hospitalization rates per 100,000. Model is augmented synthetic control models. Estimate is average treatment effect on the treated and error bars are jackknife standard errors. A = Maryland, B = Michigan, and C = Missouri. Full model highlighted in red

We provided the second sensitivity analysis, utilizing a leave-one-out approach to creating a donor pool from which to construct the synthetic control in Fig. 4. For each state, we ran the ASCM with one state omitted from the donor pool and estimated the average treatment effect on the treated and standard error. We note the full models (seen in Fig. 1) in red at the bottom of each panel. Regarding Maryland’s HPL law adoption, most models displayed similar statistical significance with roughly the same average treatment effect on the treated and standard error as the full model aside from Louisiana, which had a smaller standard error and an average treated effect on the treated slightly towards 0 but still statistically significant. For Michigan, there were also no differences when comparing significance; all models displayed non-significant estimated effects associated with HPL law repeal. Missouri’s models largely followed the same pattern, with one exception: When North Carolina was withdrawn from the model, the average treatment effect on the treated was non-significantly different (average treatment effect on the treated = 0.368, standard error = 0.359).

## Discussion

This study is the first to assess the impact of HPL laws on non-fatal firearm violence rates. We utilized a publicly available dataset from the RAND Corporation (Smart et al. 2000) derived from H-CUP data, though the outcome was not disaggregated by intent. Previous research suggested that nearly 90% of individuals attempting suicide with a firearm ultimately die from it (Azrael and Miller 2016). A large portion of our non-fatal FIH rates may then stem from non-suicide-based firearm violence, though that is not confirmable using the current dataset. Despite the data's age, it remains the most comprehensive dataset estimating non-fatal firearm violence resulting in hospitalization. Future research on HPL laws' impact should consider disaggregating outcomes by intent, although publicly accessible H-CUP data are unavailable.

We investigated the association between changes in HPL laws and FIH rates, the first examination of these laws on non-fatal firearm violence. Specifically, we focused on Missouri's repeal, Michigan's partial repeal, and Maryland's adoption of an HPL law from 2000 to 2016. The results suggest that the adoption of HPL laws was associated with a 32.3% reduction in FIH rates. In contrast, Missouri's full repeal of HPL was associated with a 35.7% increase in FIH rates. Michigan's partial repeal, where the HPL license requirement was still required for non-FFL transactions, did not exhibit any significant relationships with FIH rates.

We conducted several sensitivity analyses that suggested our findings are robust. While our in-place placebo tests show variation in effect across our placebo controls, findings from Maryland and Missouri suggested they are outliers compared to all other controls. In a similar vein, Michigan’s result was not an outlier, indicating robustness to the null findings. Our leave-one-out sensitivity analyses further support the robustness of these models against various combinations of donor pools. For Maryland and Michigan, all combinations of donor pools produced results consistent with the full donor pool models. For Missouri, when North Carolina was left out of the donor pool, the model produced an average treatment effect on the treated similar to the full model, with a larger standard error, causing it to be non-significant. This finding is logical as North Carolina supplied the largest donor weight for Missouri. We note that North Carolina is not only politically similar to Missouri (its legislature voted to repeal its HPL law in 2023), but it also contained pre-treatment outcome and covariate values that are close to that of Missouri’s values (see Supplemental Table 1 for average pre-treatment covariate values).

Our findings align with prior research on the impact of HPL laws on firearm injuries, extending the research to include non-fatal violence. Adopting a HPL law was associated with decreased rates of FIH, consistent with prior research linking adoption to lower rates of homicides and suicides in Connecticut (Rudolph et al. 2015; McCourt et al. 2020; Crifasi et al. 2015). Conversely, repealing a HPL law in Missouri was associated with increased rates of FIH, mirroring earlier findings that such repeals correlated with higher homicide rates (Hasegawa et al. 2019; Webster et al. 2014; Li et al. 2023) and suicide rates (McCourt et al. 2020; Crifasi et al. 2015; Bhatt et al. 2020). Our finding of null effects concerning Michigan's HPL law change likely stems from the law's partial repeal. Following the partial repeal, which eliminated the licensure requirement for transactions with FFLs, Michiganders seeking firearms from an FFL remained subject to federal-level background check standards. Recent research among current firearm owners indicates that 87% of those who purchased a firearm from an FFL underwent a background check. However, this percentage dropped to 55% for online acquisitions and further reduced to 23% for firearms acquired from acquaintances or friends (Miller et al. 2017). As such, the HPL law requirements were still in place for transactions least likely to undergo a background check.

Nationally representative surveys show gaps in support for HPL between firearm owners and non-gun owners (Crifasi et al. 2020; Barry et al. 2018, 2019). Multiple surveys indicate that approximately 8 out of 10 non-gun owners endorse the requirement of a license for firearm purchases. In contrast, these studies show that support among firearm owners hovered around 6 out of 10 (Crifasi et al. 2020) and increased to 75% when examining support among firearm owners who live in states where HPL laws are in place. Despite the gap between these groups, it's noteworthy that more than half of firearm owners expressed support for the concept of requiring a license before obtaining a firearm. The findings presented here, combined with existing research, suggest that HPL laws offer protection and potentially enjoy majority policy support.

### Limitations

The limitations of this study are primarily associated with the assumptions underlying the ASCM and the SCM. The ASCM’s regression model can add excessive extrapolation when the donor weights in the control pool rely heavily on one or two states. To that end, we added an ad hoc analysis demeaning our outcomes and rerunning our analyses, finding, specifically for Missouri and Michigan, that adding the ridge regression in the ASCM did not introduce significant biases related to the mean level of our outcome variable. For Maryland, the donor weights were more dispersed. This suggested it may not be appropriate to rely on results on from the ASCM, forgoing the regression’s extrapolations. However, we saw that the average treatment effect on the treated and standard error were very similar in the SCM and ASCM models for Maryland.

The accuracy of estimating the average treatment effect on the treated hinges on the model's performance, measured by the RMSE. Our models demonstrated small RMSE values, indicating a robust fit in the pre-treatment period. The ASCM and SCM also operate under the assumption of no spill-over from donor states and no anticipation effect. Assessing the assumption of no spill-over is challenging, given that firearm crime transcends state boundaries. It is plausible that some firearm injuries contributing to the FIH rate occurred within our treatment states but were committed by individuals residing outside the state, exempt from the same legal regulations governing HPL laws. While the likelihood of this scenario significantly biasing FIH rates is minimal, it is not entirely dismissible.

While Maryland and Missouri had small RMSE values, indicating strong pre-treatment fit, the difference between each state and their synthetic control changed prior to treatment, indicating some anticipation effect. This observation suggests the possibility that omitted variables, in particular policy variables, biased our findings. Specifically for Missouri, there was evidence that their change to a more permissive concealed carry weapons law in 2004 was associated with increases in fatal firearm violence (Doucette et al. 2022b). Without the ability to control for the presence of this policy change, we cannot disentangle the two policies, though our primary models and sensitivity analyses suggested FIH rates increased in the mid to late 2010s.

Evaluations of state-level policies are susceptible to selection bias, given the non-random assignment of states to treatment or control groups. We addressed this challenge through two sensitivity analyses. We employed in-place placebo testing to examine whether our findings were indicative of broader nationwide trends or aberrant. Additionally, we conducted leave-one-out sensitivity analyses on the donor pool construction to assess the potential over-dependence on donor states.

RAND Corporation estimated the mean inpatient FIH rates using national and state-level datasets. While they used advanced statistical techniques to produce estimates of hospitalization related to firearm injury, these data excludes incidents where individuals do not present to hospital or where the injury does not warrant inpatient admission. The data also did not control for repeated hospitalizations or for death after hospitalization. As a result, our outcome likely represented only a proportion of non-fatal firearm injury. Further, more years of data are needed to examine non-fatal FIH rates.

### Conclusion

This study is the first to examine the impact of changes to HPL laws on non-fatal firearm injury. Our results suggested changes to HPL affect non-fatal firearm injury rates. Specifically, the adoption of a HPL law may reduce non-fatal firearm injuries by 32.3% and the repeal of a HPL law may increase non-fatal firearm injuries 35.7%. States seeking to address rising rates of firearm violence ought to consider adopting a HPL law to strengthen their processes for purchasing a firearm.

### Supplementary Information

Supplementary Material 1

## Data Availability

This study uses publicly available outcome data provided by the RAND corporation. The data can be accessed at https://www.rand.org/pubs/tools/TLA243-3.html.

## Footnotes

1 Michigan was the first state to partially repeal their HPL law. Therefore, the only suitable controls for law change were states that retained an HPL throughout the study period.

2 These states include all states that were *not* included in the control units for the repeal analyses.

## References

[CR1] Abadie A. Using synthetic controls: feasibility, data requirements, and methodological aspects. J Econ Lit. 2021;59(2):391–425.

[CR2] Abadie A, Gardeazabal J. The economic costs of conflict: a case study of the Basque country. Am Econ Rev. 2003;93(1):113–32.

[CR3] Abadie A, Diamond A, Hainmueller J. Comparative politics and the synthetic control method. Am J Polit Sci. 2014;59(2):495–510.

[CR4] Abadie A, Vives-i-Bastida J. Synthetic controls in action; 2022. Available from: http://arxiv.org/abs/2203.06279.

[CR6] Azrael D, Miller MJ. Reducing suicide without affecting underlying mental health. In: The international handbook of suicide prevention. Wiley; 2016. pp. 637–62. 10.1002/9781118903223.ch36.

[CR7] Barry CL, Webster DW, Stone E, Crifasi CK, Vernick JS, McGinty EE. Public support for gun violence prevention policies among gun owners and non-gun owners in 2017. Am J Public Health. 2018;108(7):878–81. 29771617 10.2105/AJPH.2018.304432 PMC5993389

[CR8] Barry CL, Stone EM, Crifasi CK, Vernick JS, Webster DW, McGinty EE. Trends in public opinion on US gun laws: majorities of gun owners and non-gun owners support a range of measures. Health Aff. 2019;38(10):1727–34. 10.1377/hlthaff.2019.00576 PMC7040851 31498657

[CR9] Ben-Michael E, Feller A, Rothstein J. The augmented synthetic control method. J Am Stat Assoc. 2021;14:1–34.

[CR10] Bhatt A, Wang X, Cheng AL, Morris KL, Beyer L, Chestnut A, et al. Association of changes in Missouri firearm laws with adolescent and young adult suicides by firearms. JAMA Netw Open. 2020;3(11): e2024303. 33146733 10.1001/jamanetworkopen.2020.24303 PMC7643031

[CR11] Brady. Brady background checks; 2023. Available from: https://www.bradyunited.org/our-work/policy/brady-background-checks. Accessed 21 Oct 2023.

[CR12] Braga AA, Wintemute GJ, Pierce GL, Cook PJ, Ridgeway G. Interpreting the empirical evidence on illegal gun market dynamics. J Urban Health. 2012;89(5):779–93. 22669643 10.1007/s11524-012-9681-y PMC3462834

[CR30] Bureau of Alcohol, Tobacco, Firearms and Explosives. Brady Law. Published 2021. Available from: https://www.atf.gov/rules-and-regulations/brady-law. Accessed 6 Feb 2024.

[CR13] CDC. WISQARS-fatal injury and violence data; 2021. Available from: https://www.cdc.gov/injury/wisqars/fatal.html. Accessed 1 Oct 2023.

[CR14] Cole MA, Elliott RJR, Liu B. The impact of the Wuhan Covid-19 lockdown on air pollution and health: a machine learning and augmented synthetic control approach. Environ Resour Econ (dordr). 2020;10:1–28. 10.1007/s10640-020-00483-4 PMC7416596 32836865

[CR15] Crifasi CK, Meyers JS, Vernick JS, Webster DW. Effects of changes in permit-to-purchase handgun laws in Connecticut and Missouri on suicide rates. Prev Med. 2015;79:43–9. 26212633 10.1016/j.ypmed.2015.07.013

[CR16] Crifasi CK, Buggs SAL, Choksy S, Webster DW. The initial impact of Maryland’s Firearm Safety Act of 2013 on the supply of crime handguns in Baltimore. RSF J Soc Sci. 2017;3(5):128–40.

[CR17] Crifasi CK, Merrill-Francis M, McCourt A, Vernick JS, Wintemute GJ, Webster DW. Association between firearm laws and homicide in urban counties. J Urban Health. 2018;95(3):383–90. 29785569 10.1007/s11524-018-0273-3 PMC5993701

[CR18] Crifasi CK, Stone EM, McGinty B, Vernick JS, Barry CL, Webster DW. Differences in public support for handgun purchaser licensing. Inj Prev. 2020;26(1):93–5. 31492689 10.1136/injuryprev-2019-043405 PMC7040850

[CR19] Crifasi CK, McCourt AD, Webster DW. The impact of handgun purchaser licensing on gun violence. Baltimore: Johns Hopkins Center for Gun Violence Prevention and Policy; 2019. https://www.jhsph.edu/research/centers-and-institutes/johns-hopkins-center-for-gun-violence-prevention-and-policy/_docs/impact-of-handgun-purchaser-licensing.pdf.

[CR20] Doucette ML, Green C, Necci Dineen J, Shapiro D, Raissian KM. Impact of ShotSpotter technology on firearm homicides and arrests among large metropolitan counties: a longitudinal analysis, 1999–2016. J Urban Health. 2021;98:609–21. 33929640 10.1007/s11524-021-00515-4 PMC8566613

[CR21] Doucette ML, Ward JA, McCourt AD, Webster D, Crifasi CK. Officer-involved shootings and concealed carry weapons permitting laws: analysis of gun violence archive data, 2014–2020. J Urban Health. 2022a;99(3):373–84. 35536393 10.1007/s11524-022-00627-5 PMC9187822

[CR22] Doucette ML, McCourt AD, Crifasi CK, Webster DW. Impact of changes to concealed carry weapons laws on fatal and nonfatal violent crime, 1980–2019. Am J Epidemiol. 2022b. 10.1093/aje/kwac160. 10.1093/aje/kwac160 36104849

[CR23] Doucette ML, Crifasi CK, McCourt AD, Ward JA, Fix RL, Webster DW. Deregulation of public civilian gun carrying and violent crime: a longitudinal analysis 1981–2019. Criminology & Public Policy. 2023.

[CR24] Everytown for Gun Safety. Everytown gun law navigator. 2022. Gun Law Navigator. Available from: https://maps.everytown.org/navigator/. Accessed 28 Mar 2023.

[CR25] Giffords Law Center. Concealed Carry. 2022. Available from: https://giffords.org/lawcenter/gun-laws/policy-areas/guns-in-public/concealed-carry/. Accessed 26 Mar 2024.

[CR31] Giffords Law Center. Background Check Procedures. 2023. Available from: https://giffords.org/gun-laws/policy-areas/background-checks/background-check-procedures/. Accessed 24 Aug 2023.

[CR120] Giffords Law Center. Background Check Procedures in Michigan. 2024. Available from: https://giffords.org/lawcenter/state-laws/background-check-procedures-in-michigan/. Accessed 18 Aug 2024.

[CR26] Hasegawa RB, Webster DW, Small DS. Evaluating Missouri’s handgun purchaser law: a bracketing method for addressing concerns about history interacting with group. Epidemiology. 2019;30(3):371–9. 30969945 10.1097/EDE.0000000000000989

[CR5] Iowa Department of Public Safety. Acquire Weapon Permit. Available from: https://dps.iowa.gov/iowa-dps/acquire-weapon-permit. Accessed 5 July 2024.

[CR33] Johns Hopkins Center for Gun Violence Solutions. In Depth-Firearm Purchaser Licensing. 2024. Available from: https://publichealth.jhu.edu/center-for-gun-violence-solutions/solutions/firearm-purchaser-licensing/in-depth-firearm-purchaser-licensing. Accessed 23 Mar 2014.

[CR27] Jacoby SF, Dong B, Beard JH, Wiebe DJ, Morrison CN. The enduring impact of historical and structural racism on urban violence in Philadelphia. Soc Sci Med. 2018;199:87–95. 28579093 10.1016/j.socscimed.2017.05.038 PMC7437144

[CR28] Kalesan B, Vasan S, Mobily ME, Villarreal MD, Hlavacek P, Teperman S, et al. State-specific, racial and ethnic heterogeneity in trends of firearm-related fatality rates in the USA from 2000 to 2010. BMJ Open. 2014;4(9): e005628. 25239291 10.1136/bmjopen-2014-005628 PMC4185336

[CR29] Krivo LJ, Peterson RD, Kuhl DC. Segregation, racial structure, and neighborhood violent crime. AJS. 2009;114(6):1765–802. 19852253 10.1086/597285

[CR32] Li M, Dmall D, Ye T, Lin Y, Bebster D. Examining a hypothesized causal chain for the effects of the 2007 repeal of the permit-to-purchase licensing law in Missouri: homicide guns recovered in state and within a year of purchase. J Urban Health. 2023. 10.1007/s11524-023-00739-6. 37249820 10.1007/s11524-023-00739-6 PMC10322801

[CR34] McCourt AD, Crifasi CK, Stuart EA, Vernick JS, Kagawa RMC, Wintemute GJ, et al. Purchaser licensing, point-of-sale background check laws, and firearm homicide and suicide in 4 US States, 1985–2017. Am J Public Health. 2020;110(10):1546–52. 32816544 10.2105/AJPH.2020.305822 PMC7483089

[CR35] McGinty EE, Bicket MC, Seewald NJ, Stuart EA, Alexander GC, Barry CL, et al. Effects of state opioid prescribing laws on use of opioid and other pain treatments among commercially insured U.S. adults. Ann Intern Med. 2022. 10.7326/M21-4363. 35286141 10.7326/M21-4363 PMC9277518

[CR36] Miller M, Hepburn L, Azrael D. Firearm acquisition without background checks. Ann Intern Med. 2017;166(4):233–9. 28055050 10.7326/M16-1590

[CR37] Miller GF, Barnett SBL, Florence CS, McDavid Harrison K, Dahlberg LL, Mercy JA. Costs of fatal and nonfatal firearm injuries in the U.S., 2019 and 2020. Am J Prevent Med. 2024;66(2):195–204. 10.1016/j.amepre.2023.09.026 PMC10843794 38010238

[CR39] Rudolph KE, Stuart EA, Vernick JS, Webster DW. Association between connecticut’s permit-to-purchase handgun law and homicides. Am J Public Health. 2015;105(8):e49-54. 26066959 10.2105/AJPH.2015.302703 PMC4504296

[CR140] Schoenbaum H. Permit to buy handgun no longer required in North Carolina. AP News. Published 2023. Available from: https://apnews.com/article/pistol-permit-veto-override-north-carolina-b9d0ee55bf658ca72043bd3f706b128f. Accessed 6 Apr 2023.

[CR40] Siegel M, Goder-Reiser M, Duwe G, Rocque M, Fox JA, Fridel EE. The relation between state gun laws and the incidence and severity of mass public shootings in the United States, 1976–2018. Law Hum Behav. 2020;44(5):347–60. 33090863 10.1037/lhb0000378

[CR41] Smart R, Peterson S, Schell TL, Kerber R, Morral AR. Inpatient hospitalizations for firearm injury: estimating state-level rates from 2000 to 2016. RAND Corporation; 2021. Available from: https://www.rand.org/pubs/tools/TLA243-3.html. 10.7249/rhq-09-04-10 PMC9519112 36238005

[CR42] Webster D, Crifasi CK, Vernick JS. Effects of the repeal of Missouri’s handgun purchaser licensing law on homicides. J Urban Health. 2014;91(2):293–302. 24604521 10.1007/s11524-014-9865-8 PMC3978146

[CR43] Webster DW, McCourt AD, Crifasi CK, Booty MD, Stuart EA. Evidence concerning the regulation of firearms design, sale, and carrying on fatal mass shootings in the United States. Criminol Public Policy. 2020;19(1):171–212.

[CR44] Webster D, Vernick JS. Preventing the diversion of guns to criminals through effective firearm sales laws. In: Reducing gun violence in America: informing policy with evidence and analysis. The Johns Hopkins University Press; 2013.

[CR45] Wintemute GJ. Alcohol misuse, firearm violence perpetration, and public policy in the United States. Prev Med. 2015a;79:15–21. 25937594 10.1016/j.ypmed.2015.04.015

[CR46] Wintemute GJ. The epidemiology of firearm violence in the twenty-first century United States. Annu Rev Public Health. 2015b;18(36):5–19. 10.1146/annurev-publhealth-031914-122535 25533263

