# Supplementary material for: Firearm injury hospitalizations and handgun purchaser licensing laws: longitudinal evaluation of state-level purchaser licensure requirements on firearm violence, 2000–2016
Source: Inj Epidemiol. 2024 Aug 23;11:39. doi: 10.1186/s40621-024-00522-4 (PMC11342609; doi:10.1186/s40621-024-00522-4)
Supplement: Supplementary file 1 — Supplementary Material 1 [file 40621_2024_522_MOESM1_ESM.docx]

**Supplemental Material:** Firearm Hospitalizations and Handgun Purchaser Licensing laws: Longitudinal evaluation of state-level firearm licensure requirement on firearm violence, 2000-2016

**Supplemental Table 1:** Pre-treatment covariate values for donor states, donor weights, and augmented synthetic control values for Missouri.

|  |  | **Pre-Treatment Covariate Values** | | | | | | | | | |
| --- | --- | --- | --- | --- | --- | --- | --- | --- | --- | --- | --- |
| **State** | **Donor Weights** | **FIH rate per 100,000** | **Police Employ-**  **ment** | **Poverty Rate** | **Unemploy**  **-ment Rate** | **Percent Living in MSA** | **Population of Males** | **Percent Veteran** | **Percent Married** | **Personal Income Measure** | **Ethanol Con-sump-tion Rate** |
| Connecticut | 0.051 | 0.743 | 281.904 | 8.463 | 4.239 | 81.560 | 1.69E+06 | 10.677 | 54.860 | 25777.930 | 2.239 |
| Hawaii | -0.032 | 0.208 | 317.374 | 9.350 | 3.593 | 71.181 | 6.35E+05 | 12.869 | 52.043 | 18128.030 | 2.410 |
| Illinois | 0.373 | 1.377 | 396.620 | 11.325 | 5.545 | 86.694 | 6.16E+06 | 9.641 | 53.117 | 19434.710 | 2.298 |
| Iowa | -0.087 | 0.331 | 256.163 | 9.400 | 3.824 | 50.712 | 1.45E+06 | 11.993 | 59.182 | 16549.590 | 2.094 |
| Massachusetts | 0.052 | 0.539 | 318.559 | 10.200 | 4.614 | 98.161 | 3.09E+06 | 10.050 | 51.403 | 22899.510 | 2.517 |
| Nebraska | 0.120 | 0.550 | 271.120 | 9.688 | 3.413 | 55.010 | 8.63E+05 | 12.381 | 60.155 | 17424.790 | 2.266 |
| New Jersey | 0.000 | 0.822 | 479.463 | 8.025 | 4.733 | 99.964 | 4.17E+06 | 9.019 | 54.123 | 23318.700 | 2.268 |
| New York | -0.221 | 0.934 | 458.290 | 14.300 | 5.225 | 92.233 | 9.23E+06 | 8.138 | 48.128 | 21267.620 | 1.959 |
| North Carolina | 0.743 | 1.384 | 368.460 | 14.000 | 5.298 | 68.321 | 4.17E+06 | 11.993 | 55.287 | 16189.990 | 1.992 |
|  |  |  |  |  |  |  |  |  |  |  |  |
| **Missouri** |  | 1.537 | 327.359 | 10.938 | 5.052 | 71.024 | 2.80E+06 | 13.032 | 56.181 | 16559.680 | 2.307 |
| **Augmented Synthetic Control** |  | 1.432 | 351.808 | 12.482 | 5.273 | 71.967 | 3.57E+06 | 11.817 | 56.185 | 17171.858 | 2.165 |
|  |  |  |  |  |  |  |  |  |  |  |  |

**Supplemental Table 2:** Pre-treatment covariate values for donor states, donor weights, and augmented synthetic control values for Michigan.

|  |  | **Pre-Treatment Covariate Values** | | | | | | | | | |
| --- | --- | --- | --- | --- | --- | --- | --- | --- | --- | --- | --- |
| **State** | **Donor Weights** | **FIH rate per 100,000** | **Police Employ-**  **ment** | **Poverty Rate** | **Unemploy**  **-ment Rate** | **Percent Living in MSA** | **Population of Males** | **Percent Veteran** | **Percent Married** | **Personal Income Measure** | **Ethanol Con-sump-tion Rate** |
| Connecticut | 0.01 | 0.78 | 285.99 | 8.71 | 5.68 | 81.03 | 1.71E+06 | 9.71 | 52.77 | 26771.93 | 2.28 |
| Hawaii | -0.02 | 0.20 | 316.05 | 10.42 | 4.61 | 71.24 | 6.53E+05 | 12.03 | 50.51 | 18571.11 | 2.45 |
| Illinois | 0.58 | 1.36 | 397.79 | 12.08 | 6.92 | 87.06 | 6.21E+06 | 8.91 | 51.35 | 19473.86 | 2.32 |
| Iowa | -0.01 | 0.34 | 259.95 | 9.72 | 4.45 | 52.96 | 1.48E+06 | 11.31 | 57.46 | 17072.08 | 2.18 |
| Massachusetts | 0.00 | 0.54 | 318.68 | 10.50 | 5.60 | 98.63 | 3.13E+06 | 9.16 | 49.55 | 23480.12 | 2.52 |
| Nebraska | 0.01 | 0.65 | 272.14 | 10.05 | 3.72 | 56.68 | 8.80E+05 | 11.70 | 58.11 | 18074.15 | 2.29 |
| New Jersey | -0.01 | 0.81 | 480.62 | 8.81 | 6.19 | 99.87 | 4.21E+06 | 8.16 | 52.46 | 23527.61 | 2.30 |
| New York | -0.01 | 0.91 | 450.39 | 14.89 | 6.23 | 92.35 | 9.29E+06 | 7.43 | 46.49 | 21731.02 | 2.02 |
| North Carolina | 0.46 | 1.30 | 370.65 | 14.83 | 6.88 | 69.58 | 4.36E+06 | 11.27 | 53.35 | 16385.79 | 2.00 |
|  |  |  |  |  |  |  |  |  |  |  |  |
| **Michigan** |  | 1.439 | 291.498 | 12.546 | 7.988 | 81.953 | 4.90E+06 | 10.330 | 52.532 | 16799.060 | 2.184 |
| **Augmented Synthetic Control** |  | 1.372 | 386.048 | 13.340 | 6.967 | 79.452 | 5.45E+06 | 9.931 | 52.317 | 18118.267 | 2.174 |
|  |  |  |  |  |  |  |  |  |  |  |  |

**Supplemental Table 3:** Pre-treatment covariate values for donor states, donor weights, and augmented synthetic control values for Maryland.

|  |  | **Pre-Treatment Covariate Values** | | | | | | | | | |
| --- | --- | --- | --- | --- | --- | --- | --- | --- | --- | --- | --- |
| **State** | **Donor Weights** | **FIH rate per 100,000** | **Police Employ-**  **ment Rate** | **Poverty Rate** | **Unemploy**  **-ment Rate** | **Percent Living in MSA** | **Population of Males** | **Percent Veteran** | **Percent Married** | **Personal Income Measure** | **Ethanol Con-sump-tion Rate** |
| Alabama | 0.027 | 1.625 | 351.600 | 15.536 | 6.561 | 71.400 | 2.25E+06 | 11.613 | 52.669 | 15053.990 | 1.945 |
| Alaska | 0.052 | 0.835 | 289.298 | 9.650 | 7.083 | 46.960 | 3.53E+05 | 14.765 | 51.139 | 20740.710 | 2.759 |
| Arizona | 0.052 | 1.412 | 351.053 | 16.143 | 6.457 | 92.355 | 2.98E+06 | 12.036 | 51.194 | 15872.570 | 2.455 |
| Arkansas | -0.105 | 1.150 | 326.073 | 16.979 | 6.197 | 57.156 | 1.38E+06 | 12.019 | 54.715 | 14453.460 | 1.746 |
| California | 0.086 | 1.229 | 361.759 | 14.050 | 7.673 | 97.831 | 1.80E+07 | 7.970 | 48.726 | 20049.090 | 2.255 |
| Colorado | 0.031 | 0.666 | 363.330 | 10.729 | 5.751 | 85.931 | 2.40E+06 | 11.450 | 53.750 | 19435.440 | 2.714 |
| Delaware | 0.009 | 1.269 | 451.270 | 10.257 | 5.268 | 81.575 | 4.17E+05 | 12.059 | 51.037 | 19943.720 | 3.249 |
| Florida | -0.101 | 1.088 | 414.944 | 13.179 | 6.307 | 93.817 | 8.79E+06 | 12.128 | 52.592 | 17949.900 | 2.623 |
| Georgia | -0.052 | 1.327 | 531.403 | 14.800 | 6.506 | 79.209 | 4.49E+06 | 10.391 | 50.636 | 16539.590 | 2.056 |
| Idaho | -0.060 | 0.482 | 286.410 | 11.957 | 5.784 | 59.418 | 7.36E+05 | 12.008 | 59.505 | 14947.720 | 2.534 |
| Indiana | 0.016 | 0.987 | 287.398 | 12.264 | 6.425 | 76.463 | 3.12E+06 | 10.515 | 54.446 | 16267.630 | 2.020 |
| Kansas | 0.034 | 0.942 | 381.934 | 12.129 | 5.254 | 63.536 | 1.38E+06 | 11.145 | 57.674 | 17697.350 | 1.909 |
| Kentucky | 0.007 | 0.931 | 282.604 | 16.029 | 6.868 | 55.421 | 2.08E+06 | 10.503 | 56.080 | 14946.770 | 1.800 |
| Louisiana | 0.482 | 2.446 | 495.007 | 17.964 | 6.196 | 75.669 | 2.19E+06 | 9.998 | 48.243 | 15952.860 | 2.501 |
| Maine | -0.028 | 0.289 | 221.248 | 11.800 | 5.639 | 53.821 | 6.43E+05 | 13.421 | 54.584 | 16875.580 | 2.452 |
| Minnesota | 0.077 | 0.532 | 256.850 | 8.814 | 5.087 | 73.314 | 2.57E+06 | 10.301 | 56.085 | 19478.520 | 2.503 |
| Mississippi | -0.122 | 1.252 | 331.260 | 19.729 | 7.439 | 42.390 | 1.42E+06 | 10.133 | 48.756 | 13622.760 | 2.201 |
| Montana | -0.090 | 0.519 | 290.652 | 13.986 | 5.167 | 34.724 | 4.79E+05 | 13.890 | 57.050 | 15535.590 | 2.825 |
| Nevada | 0.132 | 1.172 | 358.730 | 11.821 | 7.307 | 88.787 | 1.26E+06 | 12.521 | 49.618 | 18099.590 | 3.475 |
| New Hampshire | -0.013 | 0.267 | 282.962 | 6.493 | 4.363 | 62.044 | 6.40E+05 | 12.325 | 55.806 | 21366.500 | 4.311 |
| New Mexico | -0.072 | 0.835 | 310.057 | 18.429 | 5.821 | 63.578 | 9.71E+05 | 12.284 | 49.613 | 14738.730 | 2.375 |
| North Dakota | -0.015 | 0.406 | 254.308 | 10.971 | 3.355 | 46.326 | 3.33E+05 | 11.210 | 56.195 | 18212.150 | 2.807 |
| Ohio | -0.032 | 1.003 | 297.960 | 12.614 | 6.724 | 80.531 | 5.60E+06 | 11.078 | 52.368 | 16861.380 | 1.986 |
| Oklahoma | 0.007 | 1.103 | 309.370 | 14.329 | 4.829 | 63.031 | 1.79E+06 | 12.437 | 54.387 | 16143.310 | 1.855 |
| Oregon | -0.040 | 0.554 | 254.999 | 12.557 | 7.556 | 77.457 | 1.83E+06 | 12.283 | 52.752 | 16694.530 | 2.487 |
| Pennsylvania | 0.141 | 1.253 | 296.035 | 11.121 | 6.038 | 84.545 | 6.09E+06 | 11.083 | 52.040 | 18814.920 | 2.146 |
| Rhode Island | 0.010 | 0.648 | 299.617 | 11.864 | 7.141 | 98.536 | 5.11E+05 | 10.190 | 48.162 | 19082.420 | 2.447 |
| South Carolina | -0.022 | 1.328 | 362.880 | 14.614 | 7.426 | 75.526 | 2.14E+06 | 12.047 | 51.050 | 14869.570 | 2.351 |
| South Dakota | 0.000 | 0.435 | 283.992 | 11.936 | 3.678 | 42.760 | 3.95E+05 | 12.157 | 56.118 | 17766.650 | 2.556 |
| Tennessee | 0.050 | 1.789 | 442.850 | 15.579 | 6.520 | 72.388 | 2.98E+06 | 10.870 | 53.158 | 16206.730 | 1.908 |
| Texas | -0.059 | 1.004 | 356.693 | 16.529 | 6.000 | 87.018 | 1.17E+07 | 9.571 | 52.795 | 17267.970 | 2.236 |
| Utah | 0.038 | 0.504 | 278.366 | 9.493 | 4.949 | 86.532 | 1.29E+06 | 8.493 | 59.798 | 14798.690 | 1.334 |
| Vermont | -0.001 | 0.311 | 382.922 | 9.421 | 4.392 | 31.820 | 3.06E+05 | 11.486 | 53.532 | 18325.170 | 2.648 |
| Virginia | 0.558 | 0.820 | 305.615 | 9.721 | 4.536 | 84.000 | 3.78E+06 | 12.854 | 53.158 | 20456.620 | 2.066 |
| Washington | 0.042 | 0.583 | 225.453 | 11.050 | 6.913 | 87.026 | 3.20E+06 | 12.745 | 53.216 | 19655.620 | 2.251 |
| West Virginia | -0.092 | 0.980 | 232.012 | 15.971 | 6.103 | 53.119 | 8.99E+05 | 12.024 | 54.879 | 14127.620 | 1.744 |
| Wisconsin | 0.010 | 0.656 | 327.741 | 10.393 | 5.871 | 71.491 | 2.77E+06 | 10.627 | 54.776 | 17803.800 | 2.911 |
| Wyoming | 0.041 | 0.530 | 384.349 | 10.057 | 4.359 | 29.845 | 2.71E+05 | 13.106 | 57.067 | 20145.990 | 2.661 |
|  |  |  |  |  |  |  |  |  |  |  |  |
| **Maryland** |  | 1.537 | 327.359 | 10.938 | 5.052 | 71.024 | 2.80E+06 | 13.032 | 56.181 | 16559.680 | 2.307 |
| **Augmented Synthetic Control** |  | 1.432 | 351.808 | 12.482 | 5.273 | 71.967 | 3.57E+06 | 11.817 | 56.185 | 17171.858 | 2.165 |
|  |  |  |  |  |  |  |  |  |  |  |  |

**Supplemental Table 4:** Results of the de-meaned time-series for the augmented synthetic control analyses

|  | **Primary Model** | **Standard error** | **De-meaned Model**  **(Standard Error)** | **Standard error** |
| --- | --- | --- | --- | --- |
| **Missouri** | 0.45636571 | 0.15488059 | 0.45636569 | 0.15488058 |
| **Michigan** | -0.07437869 | 0.12965412 | -0.07437868 | 0.12965411 |
| **Maryland** | -0.49672073 | 0.12320438 | -0.49672073 | 0.12320436 |
|  |  |  |  |  |
| **Note**: Primary model is an augmented synthetic control model whereas the de-meaned model subtracts the mean from each state and year indexed response within each panel. | | | | |
